# Evaluation of Virulence Factors *In vitro*, Resistance to Osmotic Stress and Antifungal Susceptibility of *Candida tropicalis* Isolated from the Coastal Environment of Northeast Brazil

**DOI:** 10.3389/fmicb.2016.01783

**Published:** 2016-11-15

**Authors:** Diana L. Zuza-Alves, Sayama S. T. Q. de Medeiros, Luanda B. F. C. de Souza, Walicyranison P. Silva-Rocha, Elaine C. Francisco, Maria C. B. de Araújo, Reginaldo G. Lima-Neto, Rejane P. Neves, Analy S. de Azevedo Melo, Guilherme M. Chaves

**Affiliations:** ^1^Medical and Molecular Micology Laboratory, Department of Clinical and Toxicological Analyses, Federal University of Rio Grande do NorteNatal, Brazil; ^2^Department of Mycology, Federal University of PernambucoSão Paulo, Brazil; ^3^Department of Oceanography and Limnology, Federal University of Rio Grande do NorteNatal, Brazil; ^4^Department of Tropical Medicine, Federal University of PernambucoRecife, Brazil; ^5^Department of Mycology, Federal University of Pernambuco, RecifePernambuco, Brazil

**Keywords:** *Candida tropicalis*, coastal environment, virulence factors, osmotic stress, antifungal susceptibility

## Abstract

Several studies have been developed regarding human health risks associated with the recreational use of beaches contaminated with domestic sewage. These wastes contain various micro-organisms, including *Candida tropicalis*. In this context, the objective of this study was to characterize *C. tropicalis* isolates from the sandy beach of Ponta Negra, Natal, Rio Grande do Norte, Brazil, regarding the expression of *in vitro* virulence factors, adaptation to osmotic stress and susceptibility to antifungal drugs. We analyzed 62 environmental isolates and observed a great variation among them for the various virulence factors evaluated. In general, environmental isolates were more adherent to human buccal epithelial cells (HBEC) than *C. tropicalis* ATCC13803 reference strain, and they also showed increased biofilm production. Most of the isolates presented wrinkled phenotypes on Spider medium (34 isolates, 54.8%). The majority of the isolates also showed higher proteinase production than control strains, but low phospholipase activity. In addition, 35 isolates (56.4%) had high hemolytic activity (hemolysis index > 0.55). With regard to *C. tropicalis* resistance to osmotic stress, 85.4% of the isolates were able to grow in a liquid medium containing 15% sodium chloride. The strains were highly resistant to the azoles tested (fluconazole, voriconazole and itraconazole). Fifteen strains were resistant to the three azoles tested (24.2%). Some strains were also resistant to amphotericin B (14 isolates; 22.6%), while all of them were susceptible for the echinocandins tested, except for a single strain of intermediate susceptibility to micafungin. Our results demonstrate that *C. tropicalis* isolated from the sand can fully express virulence attributes and showed a high persistence capacity on the coastal environment; in addition of showing high minimal inhibitory concentrations to several antifungal drugs used in current clinical practice, demonstrating that environmental isolates may have pathogenic potential.

## Introduction

The quality of water and sand of recreational beaches are directly linked to the conditions of sanitation, but water resources are frequent targets of clandestine sewers (dos Fernandes Vieira et al., 2013). Therefore, the pollution of beaches is a condition often associated with poor sanitation, where untreated sewage systems are dumped into the sea, and may contaminate the sand, due to the ebb and flow of tides. This could be an important risk factor for microbiological contamination for those who attend beaches. In tropical regions, the climatic conditions of heat and high humidity, together with the contact with sand, animals and poor hygiene may be related to a high incidence of superficial mycoses in recent years (Pelegrini et al., 2009).

The microbiological quality of beaches has long been evaluated only by fecal coliform bacteria found in seawater, whereas the impact that fungi may cause during environmental contamination has always been neglected (Maier et al., 2003). *Candida tropicalis* is a commensal yeast of the gastrointestinal tract of birds such as seagulls and terns, as well as fishes (Buck et al., 1977). *C. tropicalis* has also been isolated from polluted wastewater (Phaff et al., 1960), sandy beaches and coastal waters of Miami. In addition, this yeast belongs to the normal human microbiota, and has been isolated from both superficial and systemic infections (Basu et al., 2003).

*C. tropicalis* has been considered the second most frequently isolated species from episodes of candidemia in several Latin America multicentric studies and its importance as an etiologic agent of candidemia in north hemisphere countries has increased recently (Godoy et al., 2003), but the expression of virulence attributes may vary among different isolates. Adhesion to host cells is considered the first step necessary for the establishment of infection, being mediated by proteins and polysaccharides found on the cell wall of different *Candida* strains of each species (Cannon and Chaffin, 2001). According to the most recent data found in the literature, *C. tropicalis* has been described as more adherent to epithelial cells than other Non-*Candida albicans Candida* (NCAC) species (Lyon and de Resende, 2006; Biasoli et al., 2010).

Another important virulence factor of this species is the ability to form hyphae, this morphological transition is directly associated with pathogenicity (Thompson et al., 2011). *C. tropicalis* can also form biofilms. Bizerra et al. (2008) defined it as a well developed dense network of yeast cells and filamentous forms. Studies performed by several authors have reported an increased biofilm production in clinical isolates of *C. tropicalis* (Paiva et al., 2012; Pannanusorn et al., 2013; Udayalaxmi et al., 2014).

A previous study suggested the existence of a family of secreted aspartic proteinases, encoded by *SAPT* genes in the genome of *C. tropicalis*. However, only an enzyme called Sapt1p was purified from culture supernatants and biochemically characterized (Zaugg et al., 2001). *C. tropicalis* is still able to produce satisfactory amounts of phospholipase, which catalyzes the hydrolysis of phospholipids in host cells membranes (Silva et al., 2012; Mutlu Sariguzel et al., 2015). Hemolysins are another group of enzymes involved in *Candida* spp. virulence. Hemolytic activity significantly contributes to the pathogenesis of disseminated candidiasis, especially facilitating hyphal penetration (Luo et al., 2004; Tsang et al., 2007), because hemolytic factors result in the release of hemoglobin from the erythrocytes of the host for later use as an iron source (Giolo and Svidzinski, 2010).

Several virulence attributes are expressed or have their expression modulated in response to stress conditions promoted by the environment (Brown et al., 2014). *C. tropicalis* is also able to grow above 10–15% sodium chloride, which explains the reason why this species is often isolated from saline environments (Butinar et al., 2005). Halotolerance allows the prolonged survival of *C. tropicalis* at the maritime ecosystem.

Resistance of clinical isolates of *C. tropicalis* to the azoles has been extensively reported (Santhanam et al., 2013; Guinea et al., 2014; Liu et al., 2014). However, there are fewer studies related to the resistance of this species to other antifungal drugs, such as amphotericin B (Silva et al., 2012). *C. tropicalis* resistance to echinocandins has also already been described, but it is currently of low significance because of the high efficacy of these drugs and their recent adoption (Garcia-Effron et al., 2008; Eschenauer et al., 2014).

Despite the large number of investigations performed in different parts of the world with regard to microbiological aspects of coastal environments and the growing interest of society toward environmental issues, there are no current studies investigating the ability of environmental strains of *C. tropicalis* to express different virulence factors *in vitro* and susceptibility to antifungal drugs. Therefore, the present study aimed to characterize isolates of *C. tropicalis* obtained from the sand of Ponta Negra Beach, Natal, Rio Grande do Norte state, Brazil, regarding to: adhesion to human buccal epithelial cells, proteinase and phospholipase activity, biofilm formation, production of hemolysins and hypha formation. In addition, we also investigated the susceptibility of these isolates to high salt concentrations and to the following antifungal compounds: fluconazole, voriconazole, itraconazole, amphothericin B, caspofungin, micafungin, and anidulafungin.

## Materials and Methods

### Strains and Culture Conditions

We evaluated a total of 62 *C. tropicalis* isolates obtained from the sand of Ponta Negra beach, Rio Grande do Norte state, Brazil, belonging to the culture collection of the Medical and Molecular Mycology Laboratory, Department of Clinical and Toxicological Analyses, Federal University of Rio Grande do Norte. Of note, strain collections were conducted in different periods: two in the summer (March, 2012 and 2013) and a single one in the winter season (July, 2012), at six different points of the beach. The isolates were stored at -80°C in YPD liquid medium (dextrose 20 g/L, peptone 20 g/L, yeast extract 10 g/L) containing 20% glycerol. The cryotubes of 2 mL of capacity (Cralplast) were thawed on ice and 100 μL of cells suspension of each strain were added to 5 mL of YPD liquid medium and incubated in a shaker afterward (Tecnal, TE-420, São Paulo, Brazil), at 35°C for 48 h for the reactivation and verification of viability. Subsequently, 100 μL of each cell suspension was inoculated on the surface of Sabouraud Dextrose Agar (SDA; Oxoid, UK) containing 300 μg/mL of chloramphenicol (Park–Davis), using a Drigalsky loop. The plates were incubated at 37°C for 48 h. Yeast colonies were plated on CHROMagar *Candida* (CHROMagar Microbiology, Paris, France) to check for purity and screening for different color colonies. Species identification was based on the characteristics of the cells observed microscopically after cultivation on corn meal agar containing Tween 80, as well as classical methodology (Yarrow, 1998) and ID32C System (bioMerieux^®^ Marcy l’Etoile, France), whenever it was necessary. Of note, a control strain of *C. tropicalis* was used as a reference strain of the species for all the virulence attributes tested *in vitro*. In addition, two control strains of *C. albicans* (SC5314 and ATCC90028) were added for all the virulence experiments tested *in vitro*, because this species is still considered the most virulent species of the *Candida* genus (Moran et al., 2002). In addition, we randomly selected (blinded screening) 5 isolates of *C. tropicalis* obtained from patients with candidemia for comparisons.

### MALDI TOF MS Identification

*Candida tropicalis* isolates were seeded on the surface of SDA supplemented with chloramphenicol (0.05 mg/mL) at 35°C for 24 h. Proteins were extracted with formic acid according to an adapted protocol (Santos et al., 2011; Oliveira et al., 2015). Six hundred microliter of yeast cells in a concentration of 10^6^ were combined with 7 μL of formic acid 70% in a 1.5 mL micro centrifuge tube. The suspension was vortexed for 20 s and immediately transferred to a reading plate (Bruker Daltonics – USA). After evaporation, 0.5 μL of a matrix solution (10 mg/mL acid alpha-cyan-4-Hydroxycinnamicin ethanol: water: acetonitrile [1:1:1]; Sigma – USA) with 0.03% trifluoroacetic acid were added and gently mixed. The crystallization step occurred at room temperature and the isolates were analyzed in triplicate. Protein readings were performed with a Microflex LT mass spectrometer using the FlexControl 3.0 tool (Bruker Daltonics, USA). For protein profiles acquisition, we considered a mass range of 2.000 to 20.000 Da obtained in the linear mode with 40 nitrogen laser shots with variable speed rates reaching up to 60 Hz per well. Six ribosomal proteins of *Escherichia coli* were used for external calibration of protein masses analyzed, as follows: 4365.30; 5096.80; 5381.40; 6255.40; 7274.50 and 10300.10 Da. Profiling generation was performed using Biotyper 3.0 and Biotyper Real Time Classification softwares (Bruker Daltonik GmbH).

### Inoculum Standardization for *Candida tropicalis* Virulence Factors Evaluated *In vitro*

For all the virulence factors evaluated *in vitro*, the samples were initially grown in NGY medium (Difco Neopeptone 1 g/L, Dextrose 4 g/L; Difco yeast extract 1 g/L). *C. tropicalis* cells were incubated for 18–24 h in a rotatory shaker (Tecnal, TE-420, SaoPaulo, Brazil) at 30°C, 200 rpm. This culture medium produces an inoculums size of about 2 × 10^8^ cells/mL. Cultures were spectrophotometrically measured at a wavelength of 600 nm ranging from 0.8 and 1.2 (Biochrom Libra S32). Subsequently, *C. tropicalis* cells were diluted to obtain the specific inoculum needed for each attribute of virulence evaluated *in vitro* (Chaves et al., 2007).

### *Candida tropicalis* Adherence to Human Buccal Epithelial Cells (HBEC)

*Candida tropicalis* cells were grown overnight to stationary phase in NGY (0.1% Neopeptone [Difco], 0.4% glucose and 0.1% Yeast Extract [Difco]) at 30°C and were mixed with human buccal epithelial cells (HBEC) from healthy volunteers at a ratio of 10 yeast cells per HBEC. The mixtures were incubated at 37°C for 1 h with shaking; then cells were vortexed, formalin-fixed and transferred to a microscope slide. The number of *C. tropicalis* cells adhering to 150 HBEC was determined with the operator blinded to the nature of the material on the slide. Tests were done in triplicate (Bates et al., 2006).

### *Candida tropicalis* Biofilm Formation

Biofilm formation assays were performed according to Jin et al. (2003) adapted by Melo et al. (2007). At first, 100 μL aliquots of a standardized cell suspension (10^7^ cells/mL) were transferred to flat bottom 96 well microtiter plates and incubated for 1.5 h at 37°C in a shaker at 75 rpm. As controls, eight wells of each microtiter plate were handled in an identical fashion, except that no *Candida* suspensions were added. Following the adhesion phase, cell suspensions were aspirated and each well was washed twice with 150 μL of PBS to remove loosely adherent cells. A total of 100 μL of YNB medium (“Yeast Nitrogen Base”, DifcoTM) with 50 mM of glucose (D-glucose monohidratada P.A., Cinética) was added to each of the washed wells and incubated at 37°C in a shaker at 75 rpm. Biofilms were allowed to develop for 66 h and quantified by the crystal violet assay. Briefly, the biofilm-coated wells of microtiter plates were washed twice with 150 μL of PBS and then air dried for 45 min. Subsequently, each of the washed wells was stained with 110 μL of 0.4% aqueous crystal violet solution for 45 min. Afterward, each well was washed four times with 350 μL of sterile distilled water and immediately distained with 200 μL of 95% ethanol. After 45 min, 100 μL of destaining solution was transferred to a new well and the amount of the crystal violet stain in the referred solution was measured with a microtiter plate reader (SpectraMAX 340 Tunable Microplate Reader; Molecular Devices Ltda.) at 570 nm. The absorbance values for the controls were subtracted from the values for the test wells to minimize background interference. Interpretation of biofilm production was according to the criteria described by Stepanovic et al. (2007).

### Morphogenesis of *Candida tropicalis* on Solid Media

For induction of hypha formation on solid media the cells were grown in NGY. The cells were centrifuged at 3,000 *g* at room temperature and resuspended in dH2O followed by three washing steps. The inoculum size was 1 × 10^9^ cells/mL. From that suspension, 5 μL was spotted on the surface of Spider medium (Nutrient agar 10 g, Mannitol 10 g, KH_2_PO_4_ 2 g, agar 14.5 g, Distilled water 1000 mL- (Liu et al., 1994) and YPD medium containing 20% FBS fetal bovine serum (Silva-Rocha et al., 2015) (Sigma). The plates were incubated at 30°C for 7 days for subsequent observation of macromorphological aspects of the colonies. The assay was performed in triplicate. Colonies were considered fluffy if filaments could be visually observed, including at the edge of colonies, whereas wrinkled colonies showed wrinkles, but no filamentation was macroscopically observed. Smooth colonies were macroscopically absent of any kind of wrinkles or filamentation. Colony micromorphology was also observed with optical microscopy. The reference strain of *Candida parapsilosis* ATCC22019 was used as a negative control for true hyphae formation.

### *Candida tropicalis* Proteinase Production

Proteinase activity was determined by a method of Macdonald and Odds (1980). Fifty-microliter samples from NGY cultures were grown in 5 mL YCB + BSA medium (11.7 g/L Yeast Carbon Base [Difco]; 10 g/L glucose; 5 g/L bovine serum albumin, fraction V [Sigma–Aldrich]) rotated in a rotator shaker at 30°C for 72 h, 200 rpm. Proteolytic activity was determined by measuring the increase in trichloroacetic acid soluble products absorbing at 280 nm in triplicate after 1 h incubation of the culture supernatant with BSA substrate at 37°C. Specific activity was expressed as OD_280 nm_/OD_600 nm_ of the culture. OD readings equal to or below 0.02 were considered below the limit of detection of the technique and were represented as negative.

### *Candida tropicalis* Hemolysin Production

In order to evaluate hemolysin production, we followed the methodology proposed by Luo et al. (2001) with some adaptations. *C. tropicalis* cells were initially cultured on SDA at 35°C for 18 h. Strains were grown overnight in NGY broth. Ten microliters of cell culture were seeded in triplicate on the surface of SDA containing 7% fresh sheep blood (Ebe-Farma) and 3% glucose, contained in Petri dishes of 155 mm of diameter. The plates were incubated for 48 h at 37°C in an atmosphere with 5% CO_2_. After the incubation period, the presence of a clear halo around the inoculum indicated positive hemolysis. The diameter of colonies and zones of hemolysis were measured in order to obtain the hemolysis index (HI) for each strain. HI was determined by dividing the colony diameter by the precipitation zone plus colony diameter, which allowed classification of isolates in highly producers, moderate producers and low producers, according to Linares et al. (2007). As a positive control we used a beta hemolytic strain of *Streptococcus pyogenes* (Group A). The reference strain of *Candida parapsilosis* ATCC22019 was used as a negative control (Luo et al., 2001).

### *Candida tropicalis* Phospholipase Production

For detection of the phospholipase activity, the method of Price et al. (1982) was used. Overnight NGY cultures were diluted and standardized to a concentration of 2 × 10^5^ cells/mL; and the suspension of cells was inoculated in triplicate on the surface of Phospholipase agar (10 g peptone, 40 g dextrose, 16 g agar, 80 mL Egg Yolk Emulsion [Fluka] was added to 1000 mL of distilled water 1000 mL). The plates were incubated at 30°C for 72 h. After the incubation period, the diameters of the colonies and the halo formed around them were measured. The Pz (phospholipase zone) was determined by dividing the colony diameter by the precipitation zone plus colony diameter. The isolates were classified as follows, according to tertiles distribution: Pz = 1 as negative phospholipase activity; 0.82 ≤ Pz ≤ 0.88 as weak; 0.75 ≤ Pz ≤ 0.81 as moderate; 0.67 ≤ Pz ≤ 0.74 as strong phospholipase producers.

### Sensitivity of *Candida tropicalis* to Osmotic Stress in Sodium Chloride

The method of Chaves and da Silva (2012) was used with some modifications to determine the sensitivity of *C. tropicalis* to NaCl. Ten microliters volumes of NGY-grown yeast cells was transferred to 100 μL of Sabouraud Dextrose broth, with addition of NaCl (0.03–30%) in 96 wells microtiter plates [TPP, 92096], incubated at 35°C for 48 h. Growth determination was visually observed trough the turbidity perceptible within each well.

### Antifungal Susceptibility Profile of *Candida tropicalis*

Solutions of fluconazole (FLU), voriconazole (VOR), itraconazole (ITC), caspofungin (CPF), micafungin (MCF), anidulafungin (ADF) and amphotericin B (AMB) were prepared in accordance with guidelines M27-A3 (CLSI, 2008a) being diluted in RPMI 1640 (Roswell Park Memorial Institute) (Angus buffers and Biochemical, Niagara Falls, NY, USA) buffered 3-(*N*-morpholino) propanesulfonic acid (MOPS) to pH 7.0. Antifungal drugs tested were diluted serially in 10 different concentrations, namely: FLU (Pfizer Incorporated, New York, NY, USA) to 0.125–64 μg/mL; VOR (Pfizer Incorporated, New York, NY, USA), ITC (Pfizer Incorporated, New York, NY, USA), CPF (Merck, Rahway, NJ, USA), MCF (Merck, Rahway, NJ, USA), and ADF (Merck, Rahway, NJ, USA) to 0.03–16 μg/mL; and AMB (Sigma Chemical Corporation, St. Louis, MO, USA) to 0.015–8 μg/mL. The inoculum of all strains tested were obtained from 2 h cultivation in SDA at 35°C and an initial suspension prepared with 90% transmittance determined spectrophotometrically at 530 nm. Then, two serial dilutions were made, the first in saline solution (1:100) and the second in RPMI (1:20), in order to obtain final concentration of 10^3^ cells/mL. Susceptibility to antifungal agents was evaluated by broth microdilution, as recommended within document CLSI M27-A3 (CLSI, 2008a). Aliquots of 100 μL of the final inoculum solution were dispensed in microtiter plates of 96 wells containing 100 μL of various concentrations of the tested drugs. Finally, the plates were incubated at 37°C and test reading taken after 24 h incubation for echinocandins and fluconazole, and after 48h for the other azoles and AMB. Of note, we have performed readings for voriconazole at 48 h of growth, as recommended by the document M27-S4 of CLSI when 24 h growth of control is insufficient (CLSI, 2008b, 2012). All strains were tested in duplicate. MIC was defined for azoles and echinocandins to the lowest drug concentration which showed about 50% reduction in turbidity as compared to the positive control well. For AMB, the MIC was defined as the lowest concentration able to inhibit any growth visually perceptible (CLSI, 2012). In addition to the environmental isolates selected for this study, reference strains *C. tropicalis* ATCC13803, *C. parapsilosis* ATCC22019 and *C. krusei* ATCC6258 were included as control micro-organisms. The isolates were classified as resistant as the following cutoff points: ≥1 μg/mL for ITC (as recommended by the document M27-S3 of CLSI (CLSI, 2008b), VOR and echinocandins, MIC ≥ 2 μg/mL for AMB and ≥8 μg/mL to FLU (CLSI, 2012).

### Statistical Analysis

Data were analyzed using the statistical software “Graph Pad, Prism” version 6.0 and “Stata” version 11.0. Results were presented as mean + standard deviation, and differences were analyzed by the One-sample *t*-test, while the Spearman coefficient was used to assess the correlation between virulence factors. For all the analyses, *P*-values less than 0.05 were considered significant and the confidence interval of 95% was selected.

## Results

### Microbiological Profiling

We randomly selected 62 strains of *C. tropicalis*, in a manner that half of the strains were collected in the first period of the study, while the others were collected in the second period of the study (2012 and 2013, respectively). Of note, all the strains showed pure cultures and had blue color on CHROMagar *Candida*. They also presented blastoconidia, pseudo-hyphae and true hyphae on Cornmeal agar slides which contain tween 80 agar and a pattern of auxanogram and zymogram compatible with *C. tropicalis*.

### MALDI TOF MS Identification

Mass spectral fingerprints demonstrated that all *C. tropicalis* isolates were correctly identified at the species level by MALDI-TOF MS with log (score) values higher than 2.0.

### Adherence of *Candida tropicalis* to Human Buccal Epithelial Cells (HBEC)

**FIGURE 1:**
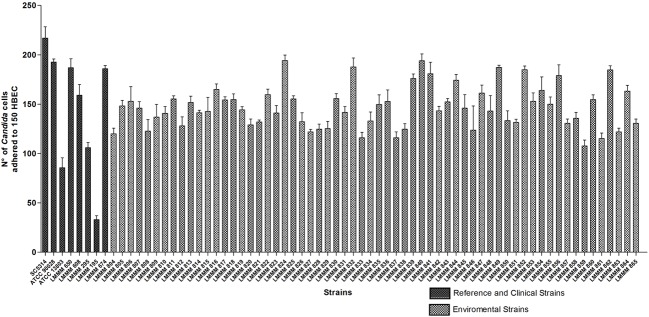
**Mean values of the number of yeast cells adhered to 150 HBEC for the environmental and clinical isolates of *Candida tropicalis. C. albicans* SC5314, ATCC90028 and *C. tropicalis* ATCC13803 reference strains.** The number of adherent yeast cells was determined by optical microscopy with 400x magnification after an incubation period of 37°C for 1 h at 200 rpm of rotation.

The ability of the isolates of *C. tropicalis* to adhere to HBEC was determined by the number of blastoconidia of each strain adhered to 150 HBEC, observed with optical microscopy. All the strains were able to adhere to HBEC. However, the isolates showed variable expression of this specific virulence factor *in vitro*. The numbers ranged from 107.7 ± 5.9 (strain LMMM859) to 194 ± 6.9 adhered cells/150 HBEC (strain LMMM840) and 194.3 ± 5.5 (strain LMMM824). All the strains tested showed an increased ability to adhere to HBEC than the control strain of *C. tropicalis* ATCC13803 (96 ± 10.0 adhered cells/150 HBEC; **Figure 1**). This difference was considered statistically significant. Most of the isolates were less adherent to HBEC than both *C. albicans* reference strains (ATCC90028; 192.7 ± 3.1 and SC5314; 217 ± 11.4; **Supplementary Table S1**). The bloodstream clinical isolates showed variable ability to adhere to the buccal epithelia. The average value for adhesion was 134.2 ± 65.4, which was considered similar to the results found for most the environmental isolates (**Figure 1**; **Supplementary Table S1**).

### Evaluation of Biofilm Formation in *Candida tropicalis*

**FIGURE 2:**
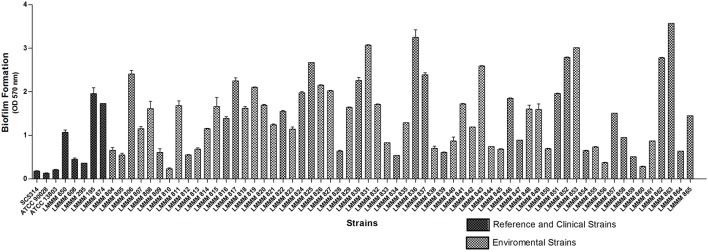
**Mean values of OD_570 nm_ of crystal violet staining of sessile cells of the environmental and clinical isolates of *C. tropicalis. C. albicans* SC5314, ATCC90028 and *C. tropicalis* ATCC13803 reference strains.** Biofilms were formed on 96-well polystyrene microtiter plates after cells growth in YNB glucose liquid medium at 37°C, during 66 h under 75 rpm of rotation.

Biofilm formation was induced in microtiter plates and quantified by spectrophotometry at 570 nm, after crystal violet staining. All the strains were able to form biofilm on polystyrene plates. Of note, a remarkable variation was observed, with the values ranging from OD570 nm of 0.23 ± 0.02 (strain LMMM810) to OD570 nm of 3.57 ± 0.00 (strain LMMM863). Forty-one strains (66.1%) were high biofilm producers (OD570 nm > 0.85), while four strains showed extremely high values of biofilm formation of OD570 nm > 3.0. Only three strains showed low production of biofilm (OD570 nm of 0.21–0.42), (LMMM810, LMMM856, and LMMM860). However, the OD570nm reading presented by *C. tropicalis* ATCC13803 (0.21 ± 0.01) was still below the lower producing isolates of our study. A statistically significant difference was observed for biofilm production of each isolate separately compared to the reference strains (*C. albicans* ATCC90028 and SC5314; *C. tropicalis* ATCC13803), except for strain LMMM810, compared to *C. tropicalis* (**Figure 2**; **Supplementary Table S1**). The mean value of bloodstream isolates optical density for biofilm formation was similar to the average obtained for the environmental isolates (1.11 ± 0.73 versus 1.45 ± 0.03, respectively), meaning that our strains are able to form biofilms as well as clinical strains isolated from episodes of candidemia.

### Evaluation of *Candida tropicalis* Morphogenesis on Solid Medium

**FIGURE 3:**
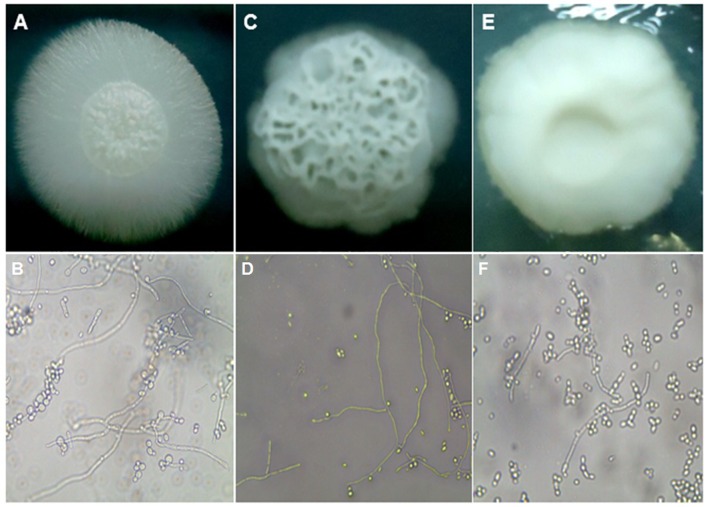
**Macro and micromorphological aspects of colonies grown on Spider medium agar, after seven days of incubation at 30°C, respectively.** **(A,B)** *C. tropicalis* ATCC13803 (fluffy phenotype); **(C,D)** *C. tropicalis* LMMM804 (wrinkled phenotype); **(E,F)** *C. parapsilosis* ATCC22019 (smooth phenotype).

In order to induce filamentation of *C. tropicalis* strains on solid medium, all the isolates were grown on the surface of plates containing Spider medium and incubated at 30°C for 7 days. As shown in S1, the majority of the isolates were classified as wrinkled (35 isolates, 56.5%). Among the others, 19 (30.6%) strains were considered of fluffy phenotype, while 8 strains (12.9%) presented a smooth phenotype, where filamentation was not observed. Microscopically, colonies classified as fluffy showed well-developed thick hyphae and pseudo-hyphae and very few blastoconidia. Wrinkled colonies also showed true hyphae, but they were thinner and shorter, while shorter pseudohyphae and blastoconidia were found in higher amounts. More than 90% of the cells of smooth colonies were blastoconidia, with a very few and short pseudohyphae found (**Figure 3**).

The same trend was found when the induction was performed on YPD + 20% FBS, although the induction of filamentation was generally more pronounced. Even colonies classified as smooth on Spider medium showed microscopically slightly more numbers of pseudohyphal cells when grown in the presence of serum (data not shown). Control strains for both species and the clinical isolates were all filamentous (**Supplementary Table S1**).

### Determination of Proteinase Production in *Candida tropicalis*

**FIGURE 4:**
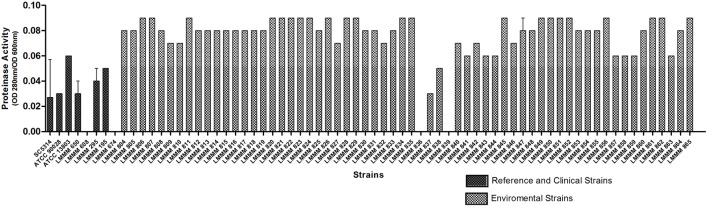
**Proteinase activity of the environmental and clinical isolates of *C. tropicalis*, *C. albicans* SC5314, ATCC90028 and *C. tropicalis* ATCC13803 reference strains as determined by the OD_280 nm_/DO_600 nm_ of cultures grown for 72 h in yeast carbon base plus bovine serum albumin liquid medium**.

Proteolytic activity was determined by the increase in trichloroacetic acid (TCA) soluble products absorbing at 280 nm in triplicate, after 1h incubation of culture supernatant with BSA substrate at 37°C. Specific activity was expressed as OD_280 nm_/OD_600 nm_ of the culture. The isolates tested showed widely varying results, with LMMM836 and LMMM839 as negative producers (OD_280 nm_/OD_600 nm_ equal to 0.02) while 22 isolates (35.5%) showed increased proteinase activity (OD_280 nm_/OD_600 nm_ equal to 0.09; **Figure 4**; **Supplementary Table S1**). When each strain was compared to the reference strains, the amount of proteinase produced was significantly higher than for the results obtained for the control strains of both species for most of the clinical isolates. For the clinical isolates, the mean value of OD_280 nm_/OD_600 nm_ was 0.04 ± 0.01, including two isolates that did not produce the enzyme. This was also considered lower that the mean value of the proteolytic activity found for the environmental isolates (**Figure 4**; **Supplementary Table S1**).

### Determination of Production of Hemolysins in *Candida tropicalis*

**FIGURE 5:**
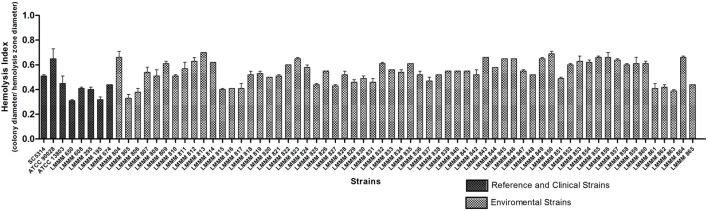
**Hemolytic activity of the environmental and clinical isolates of *C. tropicalis. C. albicans* SC5314, ATCC90028 and *C. tropicalis* ATCC13803 reference strains.** Yeast cells were grown on the surface of Sabouraud Dextrose Agar added fresh sheep blood and 3% glucose for 48 h at 37°C in an atmosphere of 5% CO_2_. The hemolytic index (HI) was determined by the ratio between the diameter of the colony and the diameter of the colony plus hemolysis zone.

In order to assess hemolytic ability, the standardized inoculum was seeded on the surface of SDA supplemented with 7% sheep blood added 3% glucose. All strains analyzed produced beta hemolysis, where HI ranged from 0.33 ± 0.03 (LMMM805, greater hemolytic activity) to 0.70 ± 0.0 (LMMM813, lower hemolytic activity). Thirty four isolates (54.8%) presented strong hemolysin production (HI ≤ 0.55), while 28 (45.2%) presented moderate production (≤0.56 HI ≤0.85). When each strain was compared to the reference strains, most of them showed similar HI of *C. albicans* ATCC90028, but were considered statistically significant different than *C. albicans* SC5314. Our environmental strains were also generally less hemolytic than *C. tropicalis* ATCC13803 and the bloodstream clinical isolates (mean value of 0.38 ± 0.06; **Figure 5**; **Supplementary Table S1**).

### Determination of Production of Phospholipase in *Candida tropicalis*

**FIGURE 6:**
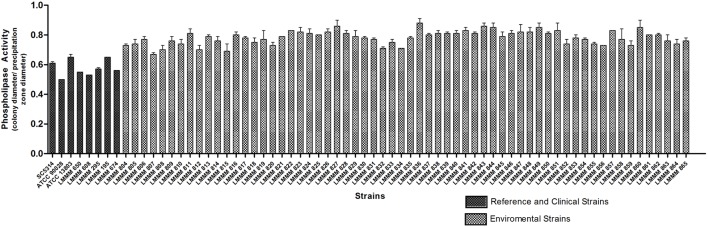
**Phospholipase activity of the environmental and clinical isolates of *C. tropicalis. C. albicans* SC5314, ATCC90028 and *C. tropicalis* ATCC13803 reference strains.** Yeast cells were grown on the surface of the phospholipase agar, after 72 h incubation at 30°C. The phospholipase zone (PZ) was determined by the ratio between the diameter of the colony and the diameter of the colony plus the precipitation halo.

Phospholipase activity was evaluated by the mean value of Pz presented by colonies on agar after incubation at 30°C for 72 h. All isolates showed positive phospholipase activity (Pz < 1). Pz values ranged from 0.67 to 0.88. The lower enzymatic activity was observed for the strain LMMM836 (Pz = 0.88 ± 0.03). Strong phospholipase production (0.67 ≤ Pz ≤ 0.74) was observed for 15 isolates (24.2%), while 32 of them (51.6%) showed moderate activity (0.75 ≤ Pz ≤ 0.81). Weak enzymatic activity was observed for 15 isolates (24.2%). Most of the isolates showed lower enzymatic activity than the reference strains of *C. albicans* (ATCC90028; Pz = 0.50 ± 0.0), SC5314 (Pz = 0.61 ± 0.01) and *C. tropicalis* ATCC13803 (0.62 ± 0.02). Our environmental strains were also generally considered lower phospholipase producers than the bloodstream isolates (mean Pz of 0.57 ± 0.05; **Figure 6**; **Supplementary Table S1**).

### Evaluation of Resistance to Osmotic Stress (Halotolerance) in *Candida tropicalis*

To evaluate resistance to osmotic stress, *C. tropicalis* cells were inoculated in Sabouraud dextrose broth with gradually increasing concentrations of NaCl. Of note, 53 strains (85.4%) were able to grow at 15% NaCl of concentration, including the reference strain *C. tropicalis* ATCC13803. The other nine environmental strains, *C. albicans* SC5314 and the clinical isolates were able to grow at 7.5% NaCl. All the strains were more resistant to osmotic stress than *C. albicans* ATCC9028, which was able to grow only in 3.75% of NaCl concentration (**Supplementary Table S1**).

### Correlation of the Virulence Factors Tested *In vitro* for All the Strains Analyzed in the Present Study

In order to verify a possible correlation among the various virulence factors studied, we have used a statistical test for obtaining the Spearman correlation coefficient, which measures the degree of linear relationship between two quantitative variables. Only a weak negative correlation between biofilm formation and the HI was observed (HI; *P* = 0.0027), meaning that the lower the biofilm production, the lower the HI, therefore the greater the hemolytic activity of these strains. For all the other attributes of virulence evaluated, no statistically significant correlation was obtained. The only other interesting finding was that 75% of filamentous colonies were also strong phospholipase producers (**Supplementary Table S1**).

### Antifungal Susceptibility Profiling of *Candida tropicalis* Isolates

Twenty-six environmental isolates of *C. tropicalis* (43.5%) were resistant to FLU that is, presented MIC values greater than 8 μg/mL after 24 h incubation. It was possible to observe the occurrence of trailing growth (“low-high” phenomenon) in five environmental isolates (8%); they were susceptible to fluconazole after 24 h incubation, but residual growth was observed at 48 h incubation, but they were considered susceptible to this drug. Another interesting phenomenon was observed for the environmental isolates against FLU; at low antifungal concentrations (0.5–2 μg/mL), some isolates showed 50% inhibition of growth compared to the positive control. However, these isolates started to grow again in the next wells, which contained higher concentrations of the antifungal agent (from 4 to 16 μg/mL) with similar growth to the positive control. This behavior is thus, similar to the paradoxical growth that occurs for echinocandins. This fact was observed in 20 environmental isolates (32.2%), and they were classified as FLU susceptible. Interestingly, a similar phenomenon also occurred for the same isolates grown in the presence of VOR. For this antifungal drug, 38 isolates (61.3%) were resistant. Regarding to ITC, resistant isolates totaled 36 (58%). It is worth to mention that 24 isolates (38.7%) were found to be susceptible dose-dependent (SDD). The atypical growth phenomenon in higher concentration of this drug previously described for FLU also occurred against ITC but only for two isolates. The number of strains resistant to the three azole tested (cross-resistance) was 15 (24.2%). Fourteen isolates (22.6%) were resistant to AMB. With respect to the echinocandins, all the strains were susceptible to the three antifungal drugs belonging to this class. Only a single isolate showed intermediate susceptibility to MCF (MIC = 0.5 μg/mL). Multidrug resistance (resistance to at least two antifungal drug classes) was observed in 12 isolates (19.3%; **Table 1**; **Supplementary Table S2**).

**Table 1:** Antifungal susceptibility profiling of *Candida tropicalis* isolates obtained from Ponta Negra beach sand, Natal, Rio Grande do Norte, Northeast Brazil.

	MIC Range (μg/mL)	GM (μg/mL)	Resistant (n/%)
Fluconazole	0.25–64	5.115	27/43.5%
Voriconazole	0.125–16	1.749	38/61.3%
Itraconazole	0.125–16	1.446	36/58%
Amphotericin B	0.125–2	0.676	14/22.6%
Caspofungin	0.03–0.25	0.045	0
Anidulafungin	0.03–0.125	0.032	0
Micafungin	0.03–0.5^∗^	0.032	0


GM, Geometric Mean; MIC, minimal inhibitory concentration. *Intermediate susceptibility.

## Discussion

The present work investigated the pathogenic potential of isolates of *C. tropicalis* obtained from the Ponta Negra beach, Natal city, Rio Grande do Norte State of Brazil.

Most of all the isolates tested were more adherent than *C. tropicalis* ATCC13803. Nevertheless, they were generally less adherent than *C. albicans* ATCC90028. These results corroborate the most current data found in the literature, where *C. albicans* is cited as the most adherent species, followed by *C. tropicalis* (Lyon and de Resende, 2006; Biasoli et al., 2010). The highly adhesive nature of the environmental isolates of the present study is consistent with data found for isolates of *C. tropicalis* in the study performed in Rio Grande do Norte by Chaves et al. (2013). Of note, the strains were isolated from the oral cavity of kidney transplant recipients, reinforcing the idea that environmental isolates may express the ability to adhere to HBEC *in vitro* as much as clinical isolates of the same species. In addition, we found in the present study that some of the environmental strains can be more adherent to the buccal epithelia than the clinical isolates from patients with candidemia.

Most of the isolates of the present study were considered strong biofilm producers. The same trend for high biofilm production in *C. tropicalis* was already reported by Tumbarello et al. (2007) in a study performed with clinical isolates obtained from hematogenic infections.

It has been reported that the fact *C. albicans* may switch between different morphologies is related to virulence (Whiteway and Bachewich, 2007). In the present study, we found a positive correlation between the ability to form hyphae and increased secretion of phospholipase. In fact Vidotto et al. (1999) verified the same correlation for isolates obtained from the oral cavity of HIV individuals, but not for the strains isolates from other body sites.

Until the present moment, there are no publications about proteinase secretion by *C. tropicalis* isolated from coastal environments. Studies report that *C. albicans* produces high levels of proteinases *in vitro*, while NCAC species show low enzymatic activity (Vidotto et al., 1999; Zaugg et al., 2001; Silva et al., 2012). This is contradictory to the results found in the present study, where the strains tested showed in general higher proteinase activity than *C. albicans* ATCC90028 and SC5314, besides the clinical isolates. The environmental stress could (with temperatures above 40°C) have somehow stimulated proteinase production. In fact, others have described that under stress, such as the presence of antifungal drugs, *C. albicans* cells affect Sap2 and Sap9 expression (Copping et al., 2005). Therefore, the high proteinase activity of the isolates of *C. tropicalis* from the beach sand is again an important finding to emphasize the possible ability of the strains to express virulence factors *in vitro*.

All the isolates analyzed in this study presented a degree of hemolytic activity, and some strains had an HI close to the values obtained for the clinical isolates, corroborating the results reported elsewhere for clinical isolates of this species (Luo et al., 2001; Rossoni et al., 2013; Riceto et al., 2015). On the other hand, most of them had lower hemolysin production than the clinical isolates. This result was expected because the clinical strains were recovered from the blood. It is in agreement with the results obtained by Favero et al. (2014) analyzing clinical isolates of *C. tropicalis* obtained from bloodstream infection.

All environmental isolates tested showed phospholipase production, contradicting what has been previously reported by Samaranayake et al. (1984). Other authors have also reported significant phospholipase activity in clinical isolates of *C. tropicalis*. According to Deorukhkar et al. (2014), such inconsistencies may be due to biological differences between isolates tested. Of note, our isolates presented low phospholipase production, unlike what was found for the reference strains and clinical isolates. It is possible that the environmental conditions did not stimulate phospholipase production, differently to what was observed for most of the virulence factors tested.

The current literature also describes that *C. tropicalis* is able to grow in culture media with a concentration above 10–15% NaCl (Butinar et al., 2005). The majority of the strains tested were resistant to a concentration up to 15% NaCl. Osmoregulation mechanisms in *C. tropicalis* are still largely unknown. It has been shown the role of ion efflux pumps in this process, emphasizing transport systems such as Na^+^/ K^+^ and Na^+^/H^+^ (Rodriguez et al., 1996; Garcia et al., 1997; Krauke and Sychrova, 2008). Therefore, it is possible that efflux pumps are over expressed by micro-organisms in the coastal environment, in response to the adaptation to stress conditions and may have somehow influenced resistance to some of the antifungal drugs tested by overexpression of efflux pumps.

In this study, we observed a remarkable number of *C. tropicalis* strains from environmental sources resistant to the azoles tested, mainly FLU. Vijaya et al. (2014) obtained very similar results, where 42.9% of isolates obtained from vaginal swabs tested were resistant to this antifungal drug. An increased number of clinical isolates of *Candida* spp. resistant to FLU have been recently reported (Figueiredo et al., 2007; Chang et al., 2013).

It is noteworthy that environmental isolates hardly have previously been exposed to antifungal compounds. However, this possibility cannot be completely ruled out, since these micro-organisms may have been derived from human fecal contamination in the coastal environment.

Some of our isolates showed the low-high phenomenon, a type of growth that is low (<2 μg/mL) after 24 h incubation, but much higher (>64 μg/mL) after 48 h (Revankar et al., 1998; Marr et al., 1999). *In vivo* studies have demonstrated that cells are actually susceptible to FLU (Rex et al., 1998). We also verified a phenomenon similar to the paradoxical growth described for *Candida* cells treated with echinocandins in our environmental strains against FLU. For echinocandins the paradoxical effect occurs as an adaptive response to the cellular damage on the fungal cell wall structure, in order to compensate for the inhibition of glucan production (Walker et al., 2010; Chen et al., 2014). Future studies using electron microscopy and ultrastructure analyses are mandatory to elucidate this phenomenon in *C. tropicalis*.

The level of resistance of the environmental isolates of *C. tropicalis* to the three azoles tested was very notorious. Jiang et al. (2013) investigating resistance to 52 clinical isolates of *C. tropicalis*, demonstrated that 18 isolates (34.6%) were resistant to FLU, while 21 (40.4%) were resistant to ITC, but only 4 (7.7%) to VOR. The authors suggest that VOR is more effective against clinical isolates of *C. tropicalis* than the other two drugs tested, which is not in agreement to our results, where 38 isolates (61.3%) are resistant to this antifungal compound. All the environmental isolates were susceptible to the echinocandins tested, even those resistant to the azoles and AMB (except for a single strain that showed intermediate susceptibility to MCF). Similar findings were observed by Castanheira et al. (2014), who reported 100% susceptibility of *C. tropicalis* blood isolates against CPF, ADF and MCF and Pfaller et al. (2015) in a surveillance study which analyzed isolates obtained from several laboratories in the Asia-Western Pacific (APAC) region Pfaller et al. (2015).

## Conclusion

This study contributed to the knowledge about the expression of virulence factors *in vitro* from *C. tropicalis*, a yeast of great prevalence in the coastal environment found in an important tourist town in northeastern Brazil. To the best of our knowledge, this was the first study to investigate the virulence of *C. tropicalis* obtained from beach sands. The significant expression of some virulence attributes and resistance to osmotic stress, leading to survival at coastal environments, suggest the potential pathogenicity of yeasts. In addition, environmental strains presented significant resistance to antifungal drugs, some with multi-drug resistance to azoles and amphotericin B. Further investigations are mandatory to elucidate the process of adaptation of this pathogen to the coastal environments and its possible correlation to the ability of colonization and infection.

## Author Contributions

DZ isolated the strains used in this study, and identified them by the classical method, made phenotypic analysis of virulence factors and prepared the manuscript. SdM greatly contributed to the experimental part. LdS did the statistical analysis. WS-R conducted the test evaluation of resistance to osmotic stress. EF and AM identified the isolates by MALDI-TOF MS. RL-N and RN did susceptibility testing to antifungal agents. GC designed all tests. All authors approved the final manuscript.

## Conflict of Interest Statement

The authors declare that the research was conducted in the absence of any commercial or financial relationships that could be construed as a potential conflict of interest.

## Abbreviations

ATCC: American type culture collection
*C. albicans*: *Candida albicans*
*C. tropicalis*: *Candida tropicalis*;
CLSI: Clinical and Laboratory Standards Institute
MALDI-TOF/MS: Matrix-assisted laser desorption time-of-flight mass
OD: optical density
