# Supplementary material for: Evaluation of Virulence Factors In vitro, Resistance to Osmotic Stress and Antifungal Susceptibility of Candida tropicalis Isolated from the Coastal Environment of Northeast Brazil
Source: Front Microbiol. 2016 Nov 15;7:1783. doi: 10.3389/fmicb.2016.01783 (PMC5108815; doi:10.3389/fmicb.2016.01783)
Supplement: Supplementary file 1 [file Table_1.DOCX]

Supplementary Material

Evaluation of virulence factors *in vitro*, resistance to osmotic stress and antifungal susceptibility of *Candida tropicalis* isolated from the coastal environment of Northeast Brazil

Diana Luzia Zuza Alves, Sayama Samara Toscano Queiroz de Medeiros, Luanda Bárbara Ferreira Canário de Souza, Walicyranison Plinio Silva-Rocha, Elaine Cristina Francisco, Maria Christina Barbosa de Araújo, Reginaldo Gonçalves Lima-Neto, Rejane Pereira Neves, Analy Salles de Azevedo Melo, Guilherme Maranhão Chaves^*^.

*** Correspondence:** Corresponding Author: email@uni.edu

# Supplementary Table

**Supplementary Table 1.**

| **Supplementary Table 1:** Virulence factors of *Candida tropicalis* isolates obtained from Ponta Negra beach sand. Natal city, Rio Grande do Norte State, Northeast Brazil | | | | | | | |
| --- | --- | --- | --- | --- | --- | --- | --- |
| **Strain number** | **Nº of *Candida* cells adhered to 150HBEC** | **Spider medium colony phenotype** | **Biofilm formation OD 570 nm** | **Proteinase activity (OD280nm/OD 600nm)** | **Hemolysis Index (colony diameter/ hemolysis zone diameter)** | **Phospholipase activity (colony diameter/ precipitation zone diameter)** | **Osmotic stress** |
| *Candida albicans* SC5314 | 217±11.4 | Fluffy | 0.18±0.01 | 0.03 ± 0.00 | 0.51 ±0.01 | 0.61±0.01 | 7.5% |
| *Candida albicans*  ATCC90028 | 192.7±3.1 | Fluffy | 0.13±0.01 | 0.03±0.00 | 0.65 ±0.06 | 0.50±0.00 | 3.75% |
| *Candida tropicalis* ATCC13803 | 96±10.0 | Fluffy | 0.21±0.01 | 0.06±0.00 | 0.45 ±0.05 | 0.65±0.02 | 15% |
| LMMM195 | 33±4 | Fluffy | 1.96±0.13 | 0.05±0.00 | 0.32±0.02 | 0.65±0.00 | 7.5% |
| LMMM295 | 106±5 | Wrinkled | 0.36±0.00 | 0.04±0.01 | 0.40±0.02 | 0.57±0.01 | 7.5% |
| LMMM608 | 159±11 | Wrinkled | 0.45±0.03 | Negative | 0.41±0.01 | 0.53±0.00 | 7.5% |
| LMMM650 | 187±9 | Fluffy | 1.07±0.05 | 0.03±0.01 | 0.31±0.01 | 0.55±0.00 | 7.5% |
| LMMM674 | 186±3 | Fluffy | 1.73±0.00 | Negative | 0.44±0.00 | 0.56±0.00 | 7.5% |
| LMMM804 | 120±5.6§*† | Wrinkled | 0.66±0.06§*† | 0.08±0.00§*† | 0.66 ±0.05§† | 0.73±0.01§*† | 15% |
| LMMM805 | 148.3±5.7§*† | Wrinkled | 0.55±0.04§*† | 0.08±0.00§*† | 0.33 ±0.03§* | 0.74±0.03§*† | 15% |
| LMMM806 | 153±14.7§*† | Wrinkled | 2.41±0.08§*† | 0.09±0.00§*† | 0.38 ±0.03§* | 0.77±0.02§*† | 15% |
| LMMM807 | 146±6.6§*† | Wrinkled | 1.15±0.05§*† | 0.09±0.00§*† | 0.54 ±0.04 | 0.67±0.01§* | 7.5% |
| LMMM808 | 122.7±11.6§*† | Fluffy | 1.61±0.17§*† | 0.08±0.00§*† | 0.51 ±0.05 | 0.70±0.03§* | 15% |
| LMMM809 | 137±12.8§*† | Fluffy | 0.61±0.08§*† | 0.07±0.00§*† | 0.61 ±0.02§† | 0.76±0.03§*† | 15% |
| LMMM810 | 140.7±7.0§*† | Fluffy | 0.23±0.02§* | 0.07±0.00§*† | 0.51 ±0.01 | 0.74±0.03§*† | 15% |
| LMMM811 | 155.3±3.2§*† | Wrinkled | 1.68±0.11§*† | 0.09±0.00§*† | 0.57 ±0.05 | 0.81±0.03§*† | 15% |
| LMMM812 | 128±9.2§*† | Wrinkled | 0.55±0.01§*† | 0.08±0.00§*† | 0.63 ±0.03§† | 0.70±0.03§* | 15% |
| LMMM813 | 151.7±6.4§*† | Smooth | 0.68±0.03§*† | 0.08±0.00§*† | 0.70 ±0.00§† | 0.79±0.01§*† | 15% |
| LMMM814 | 141.3±2.5§*† | Smooth | 1.15±0.01§*† | 0.08±0.00§*† | 0.62 ±0.00§† | 0.76±0.03§*† | 15% |
| LMMM815 | 142.7±14.2§*† | Wrinkled | 1.66±0.21§*† | 0.08±0.00§*† | 0.40 ±0.01§* | 0.69±0.05§* | 15% |
| LMMM816 | 165±5.6§*† | Fluffy | 1.39±0.04§*† | 0.08±0.00§*† | 0.41 ±0.00§* | 0.80±0.02§*† | 15% |
| LMMM817 | 154.3±3.2§*† | Fluffy | 2.25±0.07§*† | 0.08±0.00§*† | 0.41 ±0.04§* | 0.78±0.01§*† | 15% |
| LMMM818 | 154.7±5.7§*† | Wrinkled | 1.62±0.04§*† | 0.08±0.00§*† | 0.52 ±0.03 | 0.75±0.03§*† | 15% |
| LMMM819 | 144.3±3.2§*† | Wrinkled | 2.10±0.01§*† | 0.08±0.00§*† | 0.53 ±0.02 | 0.77±0.06§*† | 15% |
| LMMM820 | 129±6.0§*† | Wrinkled | 1.69±0.02§*† | 0.09±0.00§*† | 0.50 ±0.00 | 0.73±0.02§*† | 15% |
| LMMM821 | 132±2.0§*† | Wrinkled | 1.24±0.02§*† | 0.09±0.00§*† | 0.51 ±0.01 | 0.79±0.00§*† | 15% |
| LMMM822 | 159.7±5.5§*† | Smooth | 1.55±0.02§*† | 0.09±0.00§*† | 0.60 ±0.00§† | 0.83±0.00§*† | 7.5% |
| LMMM823 | 141±7.5§*† | Wrinkled | 1.14±0.05§*† | 0.09±0.00§*† | 0.65 ±0.01§† | 0.82±0.03§*† | 7.5% |
| LMMM824 | 194.3±5.5† | Wrinkled | 1.98±0.02§*† | 0.09±0.00§*† | 0.58 ±0.02§ | 0.81±0.03§*† | 15% |
| LMMM825 | 155.3±3.2§*† | Fluffy | 2.67±0.00§*† | 0.08±0.00§*† | 0.44 ±0.01§* | 0.80±0.00§*† | 7.5% |
| LMMM826 | 132.3±9.0§*† | Wrinkled | 2.15±0.01§*† | 0.09±0.00§*† | 0.55 ±0.00§ | 0.82±0.00§*† | 15% |
| LMMM827 | 122±2.6§*† | Smooth | 2.02±0.01§*† | 0.07±0.00§*† | 0.43 ±0.01§* | 0.86±0.04§*† | 15% |
| LMMM828 | 124.7±5.1§*† | Smooth | 0.64±0.02§*† | 0.09±0.00§*† | 0.52 ±0.03 | 0.81±0.02§*† | 7.5% |
| LMMM829 | 125.3±7.4§*† | Wrinkled | 1.64±0.01§*† | 0.09±0.00§*† | 0.46 ±0.02§* | 0.79±0.04§*† | 15% |
| LMMM830 | 155.7±5.0§*† | Wrinkled | 2.26±0.07§*† | 0.08±0.00§*† | 0.49 ±0.02 | 0.78±0.01§*† | 15% |
| LMMM831 | 141.7±6.0§*† | Wrinkled | 3.07±0.01§*† | 0.08±0.00§*† | 0.46 ±0.03* | 0.77±0.01§*† | 15% |
| LMMM832 | 187.7±9.1§† | Wrinkled | 1.71±0.01§*† | 0.07±0.00§*† | 0.61 ±0.01§† | 0.71±0.01§*† | 15% |
| LMMM833 | 116±5.6§*† | Fluffy | 0.83±0.00§*† | 0.08±0.00§*† | 0.56 ±0.00§ | 0.75±0.02§*† | 15% |
| LMMM834 | 133±9.0§*† | Fluffy | 0.54±0.00§*† | 0.09±0.00§*† | 0.54 ±0.02 | 0.71±0.00§*† | 15% |
| LMMM835 | 149.7±9.7§*† | Wrinkled | 1.29±0.00§*† | 0.09±0.0§*† | 0.61 ±0.00§† | 0.78±0.01§*† | 7.5% |
| LMMM836 | 152.7±11.7§*† | Wrinkled | 3.25±0.17§*† | Negative | 0.52 ±0.03 | 0.88±0.03§*† | 15% |
| LMMM837 | 116±6.0§*† | Wrinkled | 2.39±0.05§*† | 0.03±0.00† | 0.47 ±0.03* | 0.80±0.01§*† | 15% |
| LMMM838 | 124.7±5.5§*† | Wrinkled | 0.70±0.05§*† | 0.05±0.00§*† | 0.52 ±0.00 | 0.81±0.02§*† | 15% |
| LMMM839 | 176±4.6§*† | Wrinkled | 0.61±0.01§*† | Negative | 0.55 ±0.00§ | 0.81±0.01§*† | 15% |
| LMMM840 | 194±6.9§† | Wrinkled | 0.87±0.09§*† | 0.06±0.00§* | 0.55 ±0.00§ | 0.81±0.02§*† | 15% |
| LMMM841 | 180.7±11.8§† | Smooth | 1.72±0.01§*† | 0.06±0.00§*† | 0.55 ±0.00§ | 0.83±0.02§*† | 15% |
| LMMM842 | 143.3±4.5§*† | Wrinkled | 1.19±0.00§*† | 0.07±0.00§*† | 0.52 ±0.04 | 0.81±0.01§*† | 15% |
| LMMM843 | 152.3±3.5§*† | Wrinkled | 2.59±0.01§*† | 0.06±0.00§*† | 0.66 ±0.00§† | 0.86±0.02§*† | 15% |
| LMMM844 | 174.3±5.8§*† | Fluffy | 0.74±0.00§*† | 0.06±0.00§* | 0.58 ±0.00§ | 0.85±0.03§*† | 15% |
| LMMM845 | 146±13.7§*† | Fluffy | 0.68±0.01§*† | 0.09±0.00§*† | 0.65 ±0.00§† | 0.79±0.03§*† | 15% |
| LMMM846 | 123.7±24.6§*† | Fluffy | 1.85±0.01§*† | 0.07±0.00§*† | 0.65 ±0.00§† | 0.81±0.02§*† | 15% |
| LMMM847 | 161.3±8.0§*† | Fluffy | 0.89±0.00§*† | 0.08±0.00§*† | 0.55 ±0.01§ | 0.82±0.05§*† | 7.5% |
| LMMM848 | 143±15.6§*† | Smooth | 1.60±0.09§*† | 0.08±0.00§*† | 0.52 ±0.00 | 0.85±0.03§*† | 15% |
| LMMM849 | 187.3±2.1§† | Smooth | 1.59±0.13§*† | 0.09±0.00§*† | 0.65 ±0.01§† | 0.85±0.03§*† | 15% |
| LMMM850 | 133.3±10.0§*† | Wrinkled | 0.69±0.01§*† | 0.09±0.00§*† | 0.69 ±0.02§† | 0.81±0.01§*† | 15% |
| LMMM851 | 131.7±3.1§*† | Wrinkled | 1.96±0.01§*† | 0.09±0.00§*† | 0.49 ±0.01 | 0.83±0.05§*† | 15% |
| LMMM852 | 185±3.6§*† | Wrinkled | 2.79±0.01§*† | 0.09±0.00§*† | 0.60 ±0.01§† | 0.74±0.03§*† | 15% |
| LMMM853 | 153±8.5§*† | Wrinkled | 3.01±0.00§*† | 0.08±0.00§*† | 0.63 ±0.04§† | 0.78±0.02§*† | 15% |
| LMMM854 | 164±13.7§† | Fluffy | 0.65±0.01§*† | 0.08±0.00§*† | 0.62 ±0.02§† | 0.77±0.01§*† | 15% |
| LMMM855 | 150±7.2§*† | Fluffy | 0.73±0.01§*† | 0.08±0.00§*† | 0.66 ±0.01§† | 0.74±0.01§*† | 15% |
| LMMM856 | 179±10.8§† | Wrinkled | 0.37±0.01§*† | 0.09±0.00§*† | 0.66 ±0.04§† | 0.73±0.00§*† | 15% |
| LMMM857 | 130.7±4.2§*† | Wrinkled | 1.51±0.00§*† | 0.06±0.00§* | 0.64 ±0.01§† | 0.83±0.00§*† | 7.5% |
| LMMM858 | 135.7±5.9§*† | Wrinkled | 0.95±0.00§*† | 0.06±0.00§* | 0.60 ± 0.01§† | 0.77±0.07§* | 15% |
| LMMM859 | 107.7±5.9§*† | Wrinkled | 0.51±0.00§*† | 0.06±0.00§*† | 0.61 ±0.05† | 0.73±0.03§*† | 15% |
| LMMM860 | 154.7±4.7§*† | Wrinkled | 0.28±0.01§*† | 0.08±0.00§*† | 0.61 ±0.02§† | 0.85±0.05§*† | 15% |
| LMMM861 | 115.3±5.5§*† | Fluffy | 0.87±0.00§*† | 0.09±0.00§*† | 0.41 ±0.04§* | 0.80±0.00§*† | 7.5% |
| LMMM862 | 184.7±4.2§† | Fluffy | 2.78±0.01§*† | 0.09±0.00§*† | 0.42 ±0.02§* | 0.80±0.01§*† | 15% |
| LMMM863 | 122±3.6§*† | Fluffy | 3.57±0.00§*† | 0.06±0.00§* | 0.39 ±0.01§* | 0.76±0.04§*† | 15% |
| LMMM864 | 163.3±5.9§*† | Fluffy | 0.64±0.00§*† | 0.08±0.00§*† | 0.66 ±0.01§† | 0.74±0.03§*† | 15% |
| LMMM865 | 130.7±4.2§*† | Fluffy | 1.45±0.00§*† | 0.09±0.00§*† | 0.44±0.00§* | 0.76±0.02§*† | 15% |
| **MEAN** | 147.1±7.1 | - | 1.45±0.03 | 0.08±0.00 | 0.55±0.02 | 0.78±0.02 | - |
| **MEDIAN** | 145.2±5.9 | - | 1.42±0.01 | 0.08±0.00 | 0.55±0.01 | 0.79±0.02 | - |

Book of record of the Medical Mycology and Molecular laboratory. UFRN

Grey highlighted lanes represent reference strains and clinical isolates of *C. tropicalis*.

§ It shows statistically significant difference from the reference strain *C. albicans* SC5314;

* It shows statistically significant difference from the reference strain *C. albicans* ATCC90028;

† It shows statistically significant difference from the reference strain *C. tropicalis* ATCC13803.
