# Supplementary material for: Evaluation of Virulence Factors In vitro, Resistance to Osmotic Stress and Antifungal Susceptibility of Candida tropicalis Isolated from the Coastal Environment of Northeast Brazil
Source: Front Microbiol. 2016 Nov 15;7:1783. doi: 10.3389/fmicb.2016.01783 (PMC5108815; doi:10.3389/fmicb.2016.01783)
Supplement: Supplementary file 2 [file Table_2.DOCX]

Supplementary Material

Evaluation of virulence factors *in vitro*, resistance to osmotic stress and antifungal susceptibility of *Candida tropicalis* isolated from the coastal environment of Northeast Brazil

Diana Luzia Zuza Alves, Sayama Samara Toscano Queiroz de Medeiros, Luanda Bárbara Ferreira Canário de Souza, Walicyranison Plinio Silva-Rocha, Elaine Cristina Francisco, Maria Christina Barbosa de Araújo, Reginaldo Gonçalves Lima-Neto, Rejane Pereira Neves, Analy Salles de Azevedo Melo, Guilherme Maranhão Chaves^*^.

*** Correspondence:** Corresponding Author: email@uni.edu

# Supplementary Table

**Supplementary Table 2.**

| **Supplementary Table 2:** Results of susceptibility test of *Candida tropicalis* isolates from sand of Ponta Negra Beach. Natal city, Rio Grande do Norte State, Northeast Brazil | | | | | | | |
| --- | --- | --- | --- | --- | --- | --- | --- |
| **MIC values (µg/mL) for antifungal drugs tested** | | | | | | | |
| **Strain** | **FLU** | **VOR** | **ITC** | **AMB** | **CPF** | **ADF** | **MCF** |
| *C. krusei* ATCC6258 | ≥ 64 (R) | 2.0 (R) | 4 (R) | 1.0 (S) | 0.125 (S) | ≤ 0.03 (S) | ≤ 0.03 (S) |
| *C. tropicalis* ATCC13803 | 0.25 (S) | 1.0 (R) | 0.5 (SDD) | 1.0 (S) | 0.03 (S) | ≤ 0.03 (S) | ≤ 0.03 (S) |
| *C. parapsilosis* ATCC22019 | 1.0 (S) | 0.5(SDD) | 0.5(SDD) | 1.0 (S) | 0.25 (S) | ≤ 0.25 (S) | ≤ 0.03 (S) |
| LMMM804 | 1.0 (S) † | 1.0 (R) | 0.5 (SDD) | 1.0 (S) | ≤ 0.03 (S) | ≤ 0.03 (S) | ≤ 0.03 (S) |
| LMMM805 | 1.0 (S) † | 0.25 (SDD) | 4.0 (R) | 2.0 (R) | 0.03 (S) | ≤ 0.03 (S) | ≤ 0.03 (S) |
| LMMM806 | ≥ 64 (R) | 2.0 (R) | 4.0 (R) | 1.0 (S) | 0.03 (S) | ≤ 0.03 (S) | ≤ 0.03 (S) |
| LMMM807 | 1.0 (S) † | 0.125 (S) | 1.0 (R) | 1.0 (S) | ≤ 0.03 (S) | ≤ 0.03 (S) | ≤ 0.03 (S) |
| LMMM808 | 0.5 (S) † | 2.0 (R) | 1.0 (R) | 2.0 (R) | 0.03 (S) | ≤ 0.03 (S) | ≤ 0.03 (S) |
| LMMM809 | 0.5 (S) † | 4.0 (R) | 1.0 (R) | 1.0 (S) | 0.03 (S) | ≤ 0.03 (S) | ≤ 0.03 (S) |
| LMMM810 | 0.5 (S)* | 4.0 (R) | 1.0 (R) | 1.0 (S) | 0.03 (S) | ≤ 0.03 (S) | ≤ 0.03 (S) |
| LMMM811 | ≥ 64 (R) | 0.5 (SDD) | 0.5 (SDD) | 1.0 (S) | ≤ 0.03 (S) | ≤ 0.03 (S) | ≤ 0.03 (S) |
| LMMM812 | 4.0(SDD) | 2.0 (R) | 0.5 (SDD) | 2.0 (R) | 0.03 (S) | ≤ 0.03 (S) | ≤ 0.03 (S) |
| LMMM813 | ≥ 64 (R) | ≥ 16 (R) | ≥ 16 (R) | 1.0 (S) | 0.03 (S) | ≤ 0.03 (S) | ≤ 0.03 (S) |
| LMMM814 | 8.0 (R) | 2.0 (R) | 8.0 (R) | 2.0 (R) | ≤ 0.03 (S) | ≤ 0.03 (S) | ≤ 0.03 (S) |
| LMMM815 | 2.0 (S) † | ≥ 16 (R) | ≥ 16 (R) | 0.25 (S) | 0.125 (S) | ≤ 0.03 (S) | ≤ 0.03 (S) |
| LMMM816 | ≥ 64 (R) | 2.0 (R) | 2.0 (R) | 1.0 (S) | 0.125 (S) | ≤ 0.03 (S) | ≤ 0.03 (S) |
| LMMM817 | ≥ 64 (R) | 4.0 (R) | ≥ 16 (R) | 2.0 (R) | ≤ 0.03 (S) | ≤ 0.03 (S) | ≤ 0.03 (S) |
| LMMM818 | 1.0 (S) † | 2.0 (R) | 1.0 (R) | 1.0 (S) | 0.06 (S) | ≤ 0.03 (S) | ≤ 0.03 (S) |
| LMMM819 | 0.5 (S)* | 0.5 (SDD) | 2.0 (R) | 2.0 (R) | 0.03 (S) | ≤ 0.03 (S) | ≤ 0.03 (S) |
| LMMM820 | 4.0(SDD) | 0.5 (SDD) | 2.0 (R) | 0.25 (S) | 0.06 (S) | ≤ 0.03 (S) | ≤ 0.03 (S) |
| LMMM821 | 8.0 (R) | ≥ 16 (R) | 8.0 (R) | 1.0 (S) | ≤ 0.03 (S) | ≤ 0.03 (S) | ≤ 0.03 (S) |
| LMMM822 | 2.0 (S) † | 2.0 (R) | 8.0 (R) | 1.0 (S) | 0.25 (S) | 0.125 (S) | 0.06 (S) |
| LMMM823 | 2.0 (S) | ≥ 16 (R) | ≥ 16 (R) | 0.25 (S) | 0.03 (S) | ≤ 0.03 (S) | ≤ 0.03 (S) |
| LMMM824 | ≥ 64 (R) | ≥ 16 (R) | ≥ 16 (R) | 1.0 (S) | 0.06 (S) | ≤ 0.03 (S) | ≤ 0.03 (S) |
| LMMM825 | ≥ 64 (R) | 0.25 (SDD) | ≥ 16 (R) | 2.0 (R) | 0.06 (S) | ≤ 0.03 (S) | ≤ 0.03 (S) |
| LMMM826 | ≥ 64 (R) | 0.5 (SDD) | 0.25 (SDD) | 1.0 (S) | 0.03 (S) | ≤ 0.03 (S) | ≤ 0.03 (S) |
| LMMM827 | 2.0 (S) † | 0.5 (SDD) | 0.5 (SDD) | 2.0 (R) | 0.03 (S) | ≤ 0.03 (S) | ≤ 0.03 (S) |
| LMMM828 | ≥ 64 (R) | ≥ 16 (R) | 0.5 (SDD) | 1.0 (S) | 0.03 (S) | 0.06 (S) | ≤ 0.03 (S) |
| LMMM829 | 2.0 (S)* | 0.5 (SDD) | 0.5 (SDD) | 1.0 (S) | 0.06 (S) | ≤ 0.03 (S) | ≤ 0.03 (S) |
| LMMM830 | ≥ 64 (R) | ≥ 16 (R) | 0.5 (SDD) | 0.25 (S) | ≤ 0.03 (S) | ≤ 0.03 (S) | ≤ 0.03 (S) |
| LMMM831 | ≥ 64 (R) | 1.0 (R) | 0.5 (SDD) | 2.0 (R) | 0.125 (S) | ≤ 0.03 (S) | ≤ 0.03 (S) |
| LMMM832 | 4.0(SDD) | ≥ 16 (R) | 0.125 (S) † | 0.25 (S) | ≤ 0.03 (S) | ≤ 0.03 (S) | ≤ 0.03 (S) |
| LMMM833 | 4.0(SDD) | ≥ 16 (R) | 0.25 (SDD) | 0.25 (S) | 0.125 (S) | ≤ 0.03 (S) | ≤ 0.03 (S) |
| LMMM834 | 4.0(SDD) | ≥ 16 (R) | 0.25 (SDD) | 0.25 (S) | ≤ 0.03 (S) | ≤ 0.03 (S) | ≤ 0.03 (S) |
| LMMM835 | 4.0(SDD) | 8.0 (R) | 2.0 (R) | 0.25 (S) | 0.06 (S) | ≤ 0.03 (S) | ≤ 0.03 (S) |
| LMMM836 | 8.0 (R) | ≥ 16 (R) | 2.0 (R) | 0.25 (S) | ≤ 0.03 (S) | ≤ 0.03 (S) | ≤ 0.03 (S) |
| LMMM837 | ≥ 64 (R) | 0.5 (SDD) | 0.5 (SDD) | 0.25 (S) | ≤ 0.03 (S) | ≤ 0.03 (S) | ≤ 0.03 (S) |
| LMMM838 | 0.5 (S) † | 0.5 (SDD) | 0.5 (SDD) | 2.0 (R) | 0.125 (S) | ≤ 0.03 (S) | ≤ 0.03 (S) |
| LMMM839 | 4.0(SDD) | 0.125 (S) | ≥ 16 (R) | 0.25 (S) | ≤ 0.03 (S) | ≤ 0.03 (S) | ≤ 0.03 (S) |
| LMMM840 | 4.0(SDD) | 0.125 (S) | ≥ 16 (R) | 0.25 (S) | ≤ 0.03 (S) | ≤ 0.03 (S) | ≤ 0.03 (S) |
| LMMM841 | 0.5 (S) † | 0.25 (SDD) | 0.125 (S) † | 0.25 (S) | ≤ 0.03 (S) | ≤ 0.03 (S) | ≤ 0.03 (S) |
| LMMM842 | 8.0 (R) | 4.0 (R) | ≥ 16 (R) | 0.25 (S) | 0.06 (S) | ≤ 0.03 (S) | ≤ 0.03 (S) |
| LMMM843 | ≥ 64 (R) | ≥ 16 (R) | ≥ 16 (R) | 0.125(S) | ≤ 0.03 (S) | ≤ 0.03 (S) | ≤ 0.03 (S) |
| LMMM844 | ≥ 64 (R) | ≥ 16 (R) | ≥ 16 (R) | 1.0 (S) | ≤ 0.03 (S) | ≤ 0.03 (S) | ≤ 0.03 (S) |
| LMMM845 | 2.0 (S) † | 0.25 (SDD) | 0.5 (SDD) | 0.25 (S) | ≤ 0.03 (S) | ≤ 0.03 (S) | ≤ 0.03 (S) |
| LMMM846 | 8.0 (R) | ≥ 16 (R) | 1.0 (R) | 0.25 (S) | 0.06 (S) | ≤ 0.03 (S) | ≤ 0.03 (S) |
| LMMM847 | 1.0 (S)* | 0.125 (S) | 0.25 (SDD) | 1.0 (S) | ≤ 0.03 (S) | ≤ 0.03 (S) | ≤ 0.03 (S) |
| LMMM848 | 1.0 (S)* | 0.5 (SDD) | 0.5 (SDD) | 2.0 (R) | 0.25 (S) | ≤ 0.03 (S) | ≤ 0.03 (S) |
| LMMM849 | ≥ 64 (R) | ≥ 16 (R) | 1.0 (R) | 0.25 (S) | 0.06 (S) | ≤ 0.03 (S) | ≤ 0.03 (S) |
| LMMM850 | 2.0 (S) † | ≥ 16 (R) | 0.5 (SDD) | 0.25 (S) | 0.06 (S) | ≤ 0.03 (S) | ≤ 0.03 (S) |
| LMMM851 | 2.0 (S) † | 2.0 (R) | 4.0 (R) | 1.0 (S) | ≤ 0.03 (S) | ≤ 0.03 (S) | ≤ 0.03 (S) |
| LMMM852 | ≥ 64 (R) | 0.125 (S) | ≥ 16 (R) | 2.0 (R) | 0.06 (S) | ≤ 0.03 (S) | ≤ 0.03 (S) |
| LMMM853 | 0.25 (S) † | 0.25 (SDD) | 1.0 (R) | 0.5 (S) | ≤ 0.03 (S) | ≤ 0.03 (S) | ≤ 0.03 (S) |
| LMMM854 | 8.0 (R) | 0.5 (SDD) | 0.5 (SDD) | 1.0 (S) | 0.06 (S) | ≤ 0.03 (S) | ≤ 0.03 (S) |
| LMMM855 | 2.0 (S) † | 1.0 (R) | 2.0 (R) | 2.0 (R) | 0.06 (S) | ≤ 0.03 (S) | ≤ 0.03 (S) |
| LMMM856 | 2.0 (S) † | 1.0 (R) | 0.25 (SDD) | 2.0 (R) | 0.06 (S) | ≤ 0.03 (S) | ≤ 0.03 (S) |
| LMMM857 | 8.0 (R) | ≥ 16 (R) | ≥ 16 (R) | 0.25 (S) | 0.06 (S) | 0.06 (S) | ≤ 0.03 (S) |
| LMMM858 | 8.0 (R) | 8.0 (R) | 1.0 (R) | 0.25 (S) | 0.03 (S) | ≤ 0.03 (S) | ≤ 0.03 (S) |
| LMMM859 | 4.0(SDD) | 1.0 (R) | 1.0 (R) | 1.0 (S) | ≤ 0.03 (S) | ≤ 0.03 (S) | ≤ 0.03 (S) |
| LMMM860 | 0.25 (S) † | 0.5 (SDD) | 2.0 (R) | 1.0 (S) | 0.06 (S) | 0.125 (S) | 0.5 (I) |
| LMMM861 | 8.0 (R) | 4.0 (R) | 0.5 (SDD) | 0.25 (S) | 0.03 (S) | ≤ 0.03 (S) | ≤ 0.03 (S) |
| LMMM862 | 0.125 (S)† | 0.125 (S) † | 0.25 (SDD) | 0.5 (S) | ≤ 0.03 (S) | ≤ 0.03 (S) | ≤ 0.03 (S) |
| LMMM863 | 32 (R) | 0.25 (SDD) | 0.5 (SDD) | 1.0 (S) | ≤ 0.03 (S) | ≤ 0.03 (S) | ≤ 0.03 (S) |
| LMMM864 | 4.0(SDD) | 4.0 (R) | 0.5 (SDD) | 0.25 (S) | 0.25 (S) | ≤ 0.03 (S) | ≤ 0.03 (S) |
| LMMM865 | 2.0 (S) † | 0.125 (S) † | 0.5 (SDD) | 1.0 (S) | ≤ 0.03 (S) | ≤ 0.03 (S) | ≤ 0.03 (S) |

* Isolates presenting trailing growth

†Isolates presenting paradoxical growth
